# Multimodality Imaging Approach in Alzheimer disease. Part I: Structural MRI, Functional MRI, Diffusion Tensor Imaging and Magnetization Transfer Imaging

**DOI:** 10.1590/1980-57642015DN94000318

**Published:** 2015

**Authors:** Chetsadaporn Promteangtrong, Marcus Kolber, Priya Ramchandra, Mateen Moghbel, Sina Houshmand, Michael Schöll, Halbert Bai, Thomas J. Werner, Abass Alavi, Carlos Buchpiguel

**Affiliations:** 1 Department of Radiology, University of Pennsylvania School of Medicine, Philadelphia, Pennsylvania, USA.; 2 Stanford University School of Medicine, Stanford, California.; 3 Karolinska Institutet, Alzheimer Neurobiology Center, Stockholm, Sweden.; 4 Nuclear Medicine Service, Instituto do Cancer do Estado de São Paulo, University of São Paulo, São Paulo, Brazil.; 5 Nuclear Medicine Center, Radiology Institute, University of São Paulo General Hospital , São Paulo, Brazil.

**Keywords:** Alzheimer's disease, dementia, MRI, fMRI, DTI, MTI, doença de Alzheimer, ressonância magnética, *diffusion tensor imaging*, imagem de transferência magnética

## Abstract

The authors make a complete review of the potential clinical applications of traditional and novel magnetic resonance imaging (MRI) techniques in the evaluation of patients with Alzheimer's disease, including structural MRI, functional MRI, diffusion tension imaging and magnetization transfer imaging.

## Abstract (pt)

Os autores fazem uma revisão complete das potenciais aplicações clínicas de técnicas tradicional e inovadoras de ressonância magnética na avaliação de pacientes com doença de Alzheimer, incluindo ressonância magnética estrutural, técnicas funcionais de ressonância magnética, técnica de *"diffusion tensor imaging"* e imagem de transferência magnética.

## INTRODUCTION

Alzheimer disease (AD),^1^ the most common type of dementia among senile individuals, was first identified a century ago, but in last three decades there was an increasing interest in the research of its ethiopatogenesis and therapy The clinical manifestation of AD is an impairment of a broad spectrum of cognitive domains, including language and semantic knowledge, attention and executive functions, and visuoperceptual and spatial abilities. Advance neuroimaging modalities are challenging for AD diagnosis and monitoring disease progression.

The final diagnosis can definitively be confirmed when those pathological findings are seen on a postmortem autopsy. The aggregation of Aβ peptides will form the final stage of AP. The tangles are located more inside the neurons, consisted of paired helical filaments from hyperphosphorylated tau protein. Also the brain localization of these findings are different, being the AP more concentrated in the neocortex, and the tangles more in the mesial temporal structures and entorhinal initially and latter in the neocortex.

AD can be categorized according to age of onset or mode of inheritance:

1. **Early-onset AD:** This type is found in less than 10% of all AD cases. Patients are diagnosed before 65 years of age. These cases are usually familial which is entirely autosomal dominantly inherited. The familial form is mainly caused by mutagenic changes in the amyloid precursor protein (APP), the presenilin^1^ (PSEN1) and the presenilin^2^ (PSEN2) genes.
2. **Late-onset AD:** It is the most common presentation of AD. The initial detection occurs in the senile group of patients (over 65 yr) The genetic risk is associated with the presence of the apolipoprotein E (APOE) ε4 allele.

In 2011, it was suggested new diagnostic criteria and guidelines for AD.^2^ The stages of AD were divided in the following:

1. **Preclinical AD:** It was defined by measurable abnormalities in different tests in asymptomatic individuals, reflecting how AD causes modifications in the brain years before the disease can be clinically recognized. As a consequence, this guideline does not yet provide clinical criteria for diagnosing patients at this stage. Additional research into biomarkers for AD is necessary to better define this this designation.
2. **MCI due to AD:** It is defined by the very early clinical manifestations. Patients show mild memory changes that is perceived by the patient itself and family members, without compromising the patient's functional independence in daily life activities.
3. **Dementia due to AD:** This phase is defined by abnormalities in more than two cognitive domains that compromise the patient's skills to deal with the day-to-day activities.

The new criteria includes two classes of biomarkers: the ones that reflects a pathological signature and the others that reflect nerve degeneration. Among the first class of current biomarkers we found decreased cerebrospinal fluid levels of AP and the accumulation of an amyloid tracer on a dedicated PET scan. The neurodegeneration markers are increased values of tau (total plus phosphorylated) in CSF, decreased glucose concentration in temporoparietal association cortex on ^18^F-fluorodeoxyglucose (FDG) positron emission tomography (PET) scans, and brain volume decreases as measured by magnetic resonance imaging (MRI) specially in the mesial temporal cortex but also including other brain regions.

Although no therapy option has been developed to delay the disease progress or change the natural history of AD, most researchers still believe that future treatments of AD will have more chance of success if introduced at the early phases of the disease, before any significant pathological tissue damage has occurred. Early diagnosis provides patients and family members with an opportunity to become familiar with the disease course, enabling patients to better cope with the diagnosis and be able to make decisions for healthcare, social and financial planning. Thus, biomarker tests will be essential to establish early disease stages, identify patients who should receive treatment, and monitor the effects of potential treatments. In this part of the review, the roles and limitations of the biomarkers used in MRI for AD management are discussed.

## STRUCTURAL MRI

**Figure 1:**
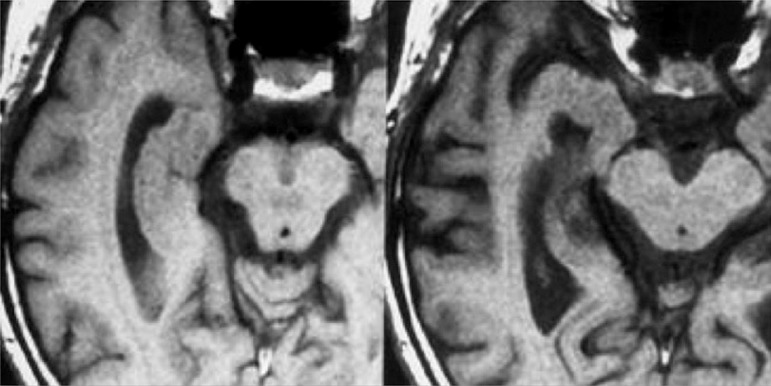
Example of structural MRI showing atrophy of the hippocampus. The right image shows a normal right hippocampus, and the left a patient with confirmed clinical diagnosis of mild cognitive decline, amnestic single domain, showing a marked atrophy of the right hippocampus.

The two most prevalent pathological features associated with dementia are cortical atrophy including medial temporal lobe atrophy and vascular changes. Structural MRI (sMRI) is important for the differential diagnosis of AD because of its ability to visualize specific atrophy patterns in the brain.^3,4^ Hippocampus atrophy, a common MRI biomarker, has been included as a key criterion for the diagnosis of AD (Figure 1).^5^

In AD is often observed continuous neuronal loss especially in the mesial temporal lobe (MTL). The entorhinal area is the first to show atrophy, and the second is the hippocampus, amygdala, and parahippocampus. It has also been shown that the posterior cingulated gyrus is also involved early in the course of AD. Atrophy is then thought to progress to other cortical and association cortical regions such as the posterior temporal and parietal cortex.^6^ By the time that typical AD patients are clinically diagnosed, atrophy is well established and prevalent in more than one brain region. This pattern of disease progression, first proposed by Braak and Braak based on studies of postmortem brain tissue has been corroborated by sMRI.

Several techniques are employed in order to differentiate those patients who have AD from either controls or those with other dementia-related diseases. Voxel-based morphometry (VBM) is a validated method for comparing volumes in brain tissue composition among groups of subjects. VBM is not restricted to one particular brain structure and gives a whole brain assessment of anatomical differences throughout the brain.^7^ Employing images as input, VBM identifies differences in brain anatomy among groups of subjects using voxel-by-voxel analysis of differences in tissue characteristics. After corrections have been made for the number of comparisons that are being performed to avoid bias, clusters of spatially-proximate voxels that meet a certain statistical threshold are highlighted into the original image.

One of the problems with the VBM approach is the fact that global versus regional effects cannot be operationalized, and the modeled effects depend upon the normalization algorithm used to compare the different brains. In other words, the particular algorithm that compares voxels affects the areas that will be deemed significant. Furthermore, the accuracy of this normalization algorithm may be entirely independent of the neurobiological differences, and thus the effects that are seen in VBM may be driven by group differences in normalization accuracy as opposed to neurobiological differences themselves.^8^ Despite the controversy surrounding VBM, many studies use these techniques to compare brain volume changes, being reproducible among various scanners including different processing approaches, as well as spatially agreeing with effects from other imaging techniques and autopsy studies.^9,10^ An alternative to the voxel-based approach is manual segmentation of Regions of Interest (ROI). VBM does not require *a priori* decisions on the regions to be analyzed, however, depending on the context it may prove more beneficial and computationally simpler to use volumes or thicknesses of particular structures as proxies for the progression of the disease. These regions should be chosen from neuropathological AD studies that aim to elucidate which brain regions are related to dementia caused by AD. The inherent problem with using ROIs is the *a priori* focus of the search for differences. With imperfect understanding of the underlying pathologies of the disease, we only see the higher-order effect of the underlying molecular mechanisms. A choice of a particular region may thus manifest differences between normal volunteers and patients at the regions downstream of underlying pathological molecular processes.

**Figure 2:**
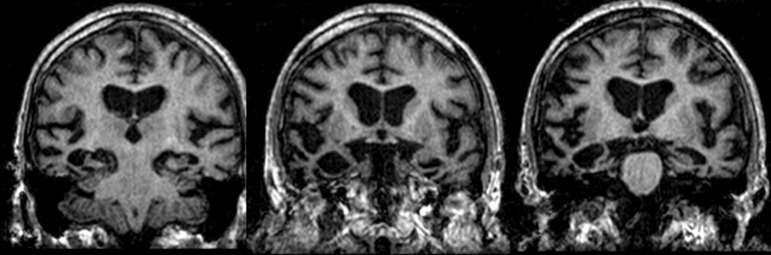
Coronal slices of a MRI scan of a patient with Alzheimer's disease. Note the marked volume reduction of both hippocampi, more intense in the right side.

Based on the framework for the progression of AD, several studies have examined the most affected cerebral regions in the very initial stage of AD. At the turn of the century, evidence began to build from postmortem autopsy studies that AD pathology is characterized by a temporospatial pattern of progressive atrophy. As evidenced by the literature, sMRI have supported the hypothesis that the MTL is one of the first areas to present decrease in volume in the progress to AD. In particular, atrophy manifests earliest in the entorhinal and perirhinal cortices of the MTL and progress from there (Figure 2).

Using a qualitative scoring of MTL atrophy could accurately differentiate AD patients from controls with specificity values ranging from 80%-85% in a memory clinic population.^11^

Although MRI images can visualize medial temporal lobe atrophy rather accurately, normal volume values do not rule out AD, and atrophy in this region is a common feature of many other neurodegenerative disorders and hence not specific for AD. Therefore, much work has gone into the specification of different regions within the medial temporal lobe where changes may foreshadow the onset of AD. Recent work has revealed that the typical pathological findings of AD, specially the neuronal loss, appear to be located most prevalently in the entorhinal cortex.^12,13^ Bobinski et al. found significant differences in the hippocampus and entorhinal cortex volumes compared to the controls studying a series of early AD patients versus a control group.^14^

Atrophy of the hippocampus in particular has been examined as a precursor to the onset of AD. Many studies have examined the efficacy of using hippocampal volume to predict the onset of dementia with mixed results. Atrophy in the hippocampus accurately differentiate patients with mild dementia from normal volunteers as well as from subjects with other neuropsychiatric diseases.^15^ Longitudinal studies following elderly patients before the manifestation of any symptoms of MCI or AD at the time of the first MRI who later developed cognitive problems and a diagnosis of MCI or AD have been able to show that volumetric reduction of MTL structures precede the finding of cognitive decline by up to 6 years.^16-18^ At the earliest, those patients exhibiting cognitive decline showed a 5% decrease in the volumes of the amygdala and the hippocampus compared to controls. Changes in MTL volume, as demonstrated by a VBM-based approach, were found to precede in years the expression of any symptoms.^19^ In a normal geriatric population at an average of 3.2 years before conversion from cognitively normal to any impairment (CDR 0 to CDR 0.5), Csernansky et al. observed changes in the hippocampus, particularly in the CA1 region.^20^

Although it has been shown that those with MCI and AD have a reduced MTL volume, the results using the hippocampus as predictive of future development of AD are inconclusive. One of the major reasons for the discrepancy of results that has been seen in the literature is the fact that there is no standardized hippocampal segmentation technique. Consequently, researchers adopt different techniques to segment the hippocampus. Current efforts are attempting to standardize the hippocampal manual tracing protocol. These standardized protocols will eventually be used as the gold standard reference for calculating hippocampus volumetry.^21^ - Another problem with the use of the hippocampus to predict AD is the fact that hippocampal volume loss, as seen on sMRI, can also be produced by disorders other than AD. As a result, the extent of brain atrophy outside of the MTL as well as the relation of that volume loss in the hippocampus is important for accurate diagnosis.^22,23^ Independent studies using ROI methods to assess hippocampal volume, however, have shown good discrimination from AD subjects and controls with 80%-90% accuracy.^8,9^ Studies using changes in hippocampal shape features have demonstrated above 90% discrimination.^10^

Studying subjects with genetic mutation linked with familial AD, Cash et al.^24^ reported GM volume changes in symptomatic carriers in the temporal lobe, precuneus, cingulate gyrus, putamen and thalamus as compared with non-carriers. WM of carriers was also lower at fornix and cingulus, projections to hippocampus, precuneus and posterior cingulate. However, no differences were observed between non-carriers and presymptomatic carriers.

sMRIs can be used with relative accuracy to differentiate AD-related dementia from other dementias. This is due to many different dementias having specific atrophy patterns that are visible on sMRIs.

For instance, besides MTL changes, reduction of volume in the parietal lobes is a common radiological finding of AD and may be helpful in differentiating from other neurodegenerative diseases associated with dementia.^11,25^ However, this is not a perfect science as there are many atypical patterns and presentations of all of the different dementias, including AD-related dementia and frontotemporal lobe (FTL)-related dementias. More basic science and applied imaging research must be done in order to more accurately use sMRIs to discern the underlying pathologies of visible brain atrophy to make sensitive and specific diagnoses.

Basic studies comparing temporal lobe volumes among healthy subjects, MCI, and AD patients described significant changes in hippocampal volume: MCI patients present around 14% decrease in size as compared to controls and AD patients around 22% reduction in size. The only different finding between AD and MCI patients was atrophy seen in the temporal neocortex in AD but not in MCI.^26^ Some studies have found that the severity of cerebral atrophy is correlated with cognitive decline, indicating that sMRI could be used to predict conversion of MCI to AD.^27^

Killiany et al.^28^ found evidence that MR quantification of brain regions that demonstrate pathological changes in the earliest stages of AD are better at differentiating patients with AD in the prodromal phase than when the same quantification is done later in the course of the disease. Furthermore, this study purports that atrophy in the more posterior portion of the anterior cingulate, begins early in the disease. When the atrophy occurs, however, is unknown. Other work has found that many early-onset AD manifestations may present a different distinct atrophy pattern predominately involving the parietal cortex, precuneus, and posterior cingulum, while the atrophy of the MTL is delayed until the advanced stages of the disease.^29,30^ sMRI studies which examine the degree of cortical and hippocampal atrophy measured by visual ratings has a strong predictive value for further cognitive decline and development of AD.^31-33^ Studies evaluating the volume changes of the entorhinal cortex and hippocampus have shown a decrease in 20-30% and 15-25%, respectively, in those affected with mild AD.^34-36^ Furthermore, volumes of both the hippocampus and entorhinal cortex predict future conversion to AD in individuals with MCI at accuracy rates between 80-85%.^37-39^ At the MCI stage, use of the entorhinal cortex volume as opposed to hippocampal volume may prove superior in prediction of progression of MCI to AD.^40,41^ These studies' findings have been refuted by one large multicenter study that showed no added benefit to using entorhinal cortex versus hippocampus.^42^ Adding to the difficulties of using hippocampal volume as a marker for conversion to AD is the false-negative rate of around 30% that was found in the ADNI cohort.^43^

Early atrophic changes in the MTL on MCI patients showed by automated data-driven methods, in particular VBM-based analyses, showed to be a strong predictor factor for conversion to AD.^44^ These patients also show greater atrophy in temporoparietal neocortex and posterior cingulated/precuneus.^42,45^ One possible confounding factor in these studies is the fact that normal aging also promotes widespread brain volume loss. However, for the most part, the location and the magnitude of the atrophy is in a different pattern from the pathology of AD.^46^

**Limitation.** In assessing dementia using sMRI, especially degeneration as a result of AD, early stages may not be as specific as PET imaging, which is able to reveal glucose hypometabolism in each of the regions associated with atrophy. Similarly, advanced and quantitative imaging modalities, such as PET and quantitative MRI techniques, may provide more insight as to the precursors and earliest stages of AD and dementia. Furthermore, sMRI does not seem to provide any additional diagnostic insight into the progression of MCI to AD when trying to diagnose the progression from MCI to AD.^47^ This is evidenced by Richard et al. who examined the efficacy of adding structural MRIs to a brief memory test in the accurate diagnosis of the progression of MCI to AD and found no significant increases in the accuracy of diagnosis.

Currently, hippocampus atrophy is seen as the best biomarker for both the diagnosis of MCI and AD as well as the conversion from MCI to AD. This, however, may soon be replaced by more quantitative techniques. While much literature has shown that sMRI is suitable for distinguishing those with AD and MCI from controls, these have been in a largely artificial setting where a cohort is chosen based on a clinical diagnosis. More work must be done to see if these techniques are viable in a true clinical situation and can be adopted by the larger medical community. One major hurdle that sMRI faces as a standalone modality for MCI and AD is sensitive and specific techniques that would be able to differentiate those with AD from other dementias, as well as the prediction from MCI to AD. In summary, sMRI is very adequate as a diagnostic and prognostic biomarker because changes observed in MRIs are parallel to the pathophysiologic changes of AD. It must be noted that the interpretation of imaging findings is *always* founded upon assumptions, whether correct or not, of the mechanisms of the diseases. So, as more is understood into the cellular pathology of AD, the better the inferences that can be made from images, especially sMRI.

## FUNCTIONAL MAGNETIC RESONANCE IMAGING: BOLD SIGNAL AND ASL MRI

Many researches have demonstrated functional alterations in brain regions, most notably in the hippocampus and MTL, while memory tasks are applied to AD and MCI patients, and in healthy APOE ε4 carrier (high risk for AD). Early functional magnetic resonance imaging (fMRI) researches in AD and MCI used memory tests and focused towards an activation pattern on fMRI. Some studies have shown consistent findings of decreased fMRI activation in MTL in AD group^48-54^ and increased MTL activation in MCI group^48,53-55^ as compared to normal volunteers. One hypothesis for that MTL hyperactivation in MCI could be compensatory mechanisms of reducing cognitive deficits that precedes the subsequent functional deterioration as patients convert to AD.^48,56^

Recent meta-analysis by Schwindt et al.^55^ found lower activation in frontal and mesial temporal lobes in AD using encoding and retrieval paradigms as compared to controls. AD subjects also showed increased functional activation in the ventral lateral prefrontal cortex that may be related to compensatory changes.

For MCI patients, it has been suggested that the increased activation at baseline may predict a rapid cognitive deterioration. In a study by Miller et al.,^49^W it was found a strong positive correlation between hippocampal activation on a visual scene encoding task during fMRI with the degree and rate of subsequent cognitive decline, by following 25 patients with MCI up to 4 years (p <0.05). Recent study by O'Brien^50^ found that subjects with CDR 0.5 at baseline showed reduced functional activation on fMRI in the right hippocampus over 2 years of clinical follow up, a finding not replicated in subjects with CDR equal to 0 (p<0.001). Moreover, they found that a faster cognitive decline was strongly associated with the degree of hippocampal functional deficit, even when correcting for age, hippocampal volume and APOE status.

Although there are consistent fMRI results among studies with clinically AD patients and MCI patients, studies in subjects with genetic risks for AD are somewhat discordant findings. Some studies have been described decrease in MTL activation.^51,57^ In contrast, various reports have showed increase in MTL activation in cognitive intact subject bearing genetic risk for AD.^54,58,59^

Both MCI and AD patients have showed impaired intrinsic functional connectivity in the default mode network using resting state functional connectivity MRI (fc-MRI). The area that more frequently showed connectivity impairment was the posterior cingulate gyrus, precuneus and prefrontal cortex, which are important components of the above mentioned network. Disruption of connectivity between hippocampus and posterior cingulum in AD patients has been proposed by Greicius et al.^53^ by showing reduced resting state activity in the above-mentioned regions on fc-MRI studies. Sorg et al.^54^ showed compromise of the connectivity in the same areas in MCI compared to controls. They also reported other affected regions in MCI at right prefrontal cortex as well as bilateral superior parietal lobes and bilateral inferior frontal gyri compared to controls. Koch et al.^58^ found lower spatial extent of coactivated areas of the anterior cingulum and parietal lobe in MCI as compared with healthy subjects, while AD patients showed lower coactivations of most default mode network regions as compared to controls. Better characterization of the connectivity impairment and the underlying neural synchrony in AD might enhance the comprehension of some clinical aspects of that kind of dementia. Those changes have been considered specific enough for distinguishing normal elderly from AD patients by some authors that could eventually be used as potential biomarker for in vivo confirmation of AD risk.^59^

Fleisher et al.^60^ suggested resting fc-MRI may be more readily applied to at-risk populations than task fMRI. They have assessed the ability of resting state fc-MRI compared with encoding signal in normal-cognitive subjects with family history of AD and at least one copy of APOE ε4 allele compared to non-APOE ε4 allele carrier subjects plus no family history of AD. During specific encoding, no regions of activation could be identified that were different in the high-risk group. However, the differentiation of the two groups could be possible with the resting state analysis that depicted nine regions in the prefrontal, orbital frontal, temporal, and parietal lobes. Therefore, encoding techniques were much less effective (effect size of 1.39) that resting state analysis (effect size of 3.35) to differentiate groups of risk. The advantages of resting state fMRI as compared to task fMRI include no performance related variability seen in activation fMRI. Resting state is a less complex methodology to conduct and to standardize. Studying on subjects with PSEN1, PSEN2 and APP, Chhatwal et al.^61^ found functional disruption of default mode network in mutation carriers before clinical symptom occurred and worsening with impairment progression.

Brain perfusion can be obtained by arterial spin labeling (ASL), and several investigators have reported ASL MRI findings in AD. It has been reported perfusion deficits in the posterior cingulum, precuneus, inferior parietal, and lateral prefrontal cortex.^62-65^ GM atrophy inducing in reduced ASL signal were taken into account in some studies, which applied atrophy correction^63,64,66^ and found remaining effects in these regional hypoperfusion. The findings seen on ASL are correlated with regional hypometabolism on FDG PET in patients with AD. Direct comparison studies^67,68^ between FDG PET and ASL MRI showed good agreement between hypometabolism and hypoperfusion pattern, more evident in bilateral angular gyrus and posterior cingulate cortex. Musiek et al.^68^ also reported very good concordance between ASL MRI and FDG PET, with area under ROC curves of 0.90 for FDG PET (95% CI 0.79-0.99) and 0.91 for ASL MRI (95% CI 0.80-1.0). It was also found that AD patients present with 30.1% lower mean whole brain CBF compared to controls by ASL MRI. However, there is some discordance between two modalities in MTL regions. Alsop et al.^69^ found areas of increased perfusion after atrophic correction in hippocampus, parahippocampus, polar portion of the temporal lobe, superior temporal and anterior cingulate. These findings are discordant with FDG PET findings of medial temporal lobe (MTL) hypometabolism. Factors such as limited spatial resolution and sensitivity to magnetic field variation could make the variance of ASL higher in the temporal lobe.^62^ ASL studies^65,70,71^ in MCI population have reported similar AD perfusion patterns, but with lesser extent in direct comparative studies. Individuals with normal cognition who carry APOE ε^4^ allele showed higher resting CBF in the MTL relative to their non-ε4 allele counterparts, while MCI patients showed decrease resting CBF compared to normal individuals.^72^ Preclinical AD may involve increases in resting CBF in an effort to compensate for metabolic alteration.

**Limitation**. There are some advantages in assessing AD using fMRI. In particular, because of it is noninvasiveness, fMRI can be done multiple times during the course of the disease. However, significant challenges still exist in performing longitudinal fMRI in neurodegenerative disease. fMRI depends deeply on critical data recording and processing. This technique can yield problems in evaluating patients with severe cognitive impairment. The imaging quality can be affected by any degree of head motion and different task response between groups can provide erroneous functional interpretation. If the patients cannot perform the task in an adequate manner, interpretation will be unreliable. Therefore, resting state fMRI is more suitable in patients with more advanced dementia. It is also important to accomplish complete further test-retest validation. BOLD response is heterogeneous across subjects and few studies^73^ evaluating fMRI activation reproducibility in cognitive impaired subjects have been reported up to now. As might be a non-negligible cross over effect between AD risk factors and the ones secondary to the experimental task in fMRI, it is mandatory to develop special strategies to use that imaging technique with the diagnostic and stratification purposes in AD.^59^

ASL MRI is a noninvasive technique and does not involve exposure to intravenous contrast media, ionizing radiation, or radioactive tracer. However, there are some limitations. Its sensitivity is limited for routine clinical application due to a low intrinsic perfusion signal-to-noise ratio compared to other methods, such as FDG PET, HMPAO SPECT or dynamic contrast enhancement MRI. ASL MRI is also unable to correct whole brain data. These limitations could affect perfusion quantification and have an effect on the perfusion status of the individual variability, which can interfere with the statistical validation. Physiologic properties should be aware and take into account when interpreting image as they can influence ASL perfusion. Another limitation that must be considered is partial volume effect. Appropriate methodology for correction should be studied.

## DIFFUSION TENSOR IMAGING

One longitudinal study carried out by Selnes et al.^74^ was able to demonstrate the predictive value of DTI for cognitive decline as well as atrophy of the MTL through the use of three parameters: fractional anisotropy (FA), radial diffusivity (DR), and mean diffusivity (MD). WM abnormalities in the MTL are not an unspecific finding and could be related to the latter stages of AD pathology; this, however, needs to be demonstrated in studies that examine the ability for DTI WM changes to differentiate those with AD from other dementias. Furthermore, these studies need more work to elucidate age-related diffusivity changes, as it has been shown that diffusivity increases with age.^75^ For instance, from childhood to adolescence, apparent diffusion coefficient (ADC) is reduced and FA is increased,^76^ and in the aging brain ADC is increased and FA is reduced.^77^ However, one limitation regarding these studies is that they are cross-sectional. More work must be done, especially longitudinal studies, to elucidate more of the way in which the brain changes over time in both normal and diseased states.

Studies focusing on MD in subjects with AD and MCI have shown elevated MD in different brain regions of patients with AD, including frontal,^78-80^ temporal,^78-84^ parietal,^80,83-86^ and occipital lobes;^79,85^ other studies however, examining the same regions, found no significant changes in MD in the frontal,^83,85,87^ parietal,^85,87^ and occipital lobes,^78,80-83,85,87^ corpus callosum,^82,85,86,88^ posterior cingulum,^89^ and temporal lobe.^80^ Studies have also identified differences in MD between controls and AD in the splenium of the corpus callosum;^80,90,91^ however, this finding was not replicated in other parts of the corpus callosum and around the posterior and anterior limb of the internal capsule.^80,87^ A study which found significant changes in the splenium has also reported a difference in the limbs of the internal capsules.^90^ A very recent paper by Li et al.^92^ focused on using DTI to examine the differences in GM between those with early-stage AD and controls. The researchers found that MD values of bilateral hippocampus, pallidum, right thalamus and caudate were significantly increased in those with those with AD. These findings show how MD cannot be reasonably used in isolation in order to determine those who have AD. In all, studies examining MD using DTI have led to largely contradictory results.

Likewise with MD, FA measurements in cognitive-impaired subjects have led to conflicting results. For instance, decreased FA has been reported in frontal lobe studies,^78-80,87,93-95^ however other studies have found no significant decrease.^82,83,85,96^ In a work that has examined FA values in the parietal lobe,^78,79,82,83,93,94^ it was not found significant differences between AD, MCI, and controls, likewise in the occipital lobe, no significant difference in FA has been reported.^80,82,83,85,87,93^ In the temporal lobe, some studies found decreased FA in those with MCI and AD as compared to controls.^80,94^ A couple studies now have demonstrated a reduction in FA in the fornix and the anterior cingulated.^84,95,97^ Others have shown a decrease in FA in the splenium of corpus callosum in AD.^80,87^

A meta-analysis conducted by Sexton et al., described reduced FA in AD patients in the majority of brain regions, with the exception of parietal WM and internal capsule.^98^ They also noted no significant differences in DTI parameters when comparing neurodegeneration of WM between hemispheres in patients with CDR 0.5 and 1.0. Changes in FA and MD were noted in the splenium and the genu of corpus callosum of AD patients, with the changes in the splenium appearing more significant. Despite the conclusions of this meta-analysis, there are many inconsistencies across publications. FA and MD changes have been noted not only with those in AD, but also those with MCI.

While there is a lot of discrepancy in the use of FA and MD, assessing changes in the genu of the corpus callosum may be helpful to distinguish MCI from controls. Longitudinal studies following those changes are necessary to determine their efficacy to diagnose AD and to track progression of MCI to AD. In all, due to the variety of techniques and different methods to calculate MD and FA, there is no clear consensus on the application of FA and MD measures to the diagnosis of MCI and AD. There are lines of research which may prove useful in the future, for instance using FA or MD measures in conjunction with other modalities to give more accurate and reliable results.

Besides assessing WM, DTI has been utilized to look at the difference in diffusion characteristics, which is believed to be caused by specific damage such as demyelination, intra-axonal changes, and neuronal loss. Hanyu et al.^99^ examined the diffusion patterns within the corpus callosum in patients with AD. They found reduced anisotropy in the genu and splenium of corpus callosum, suggesting the presence of axonal loss as well as demyelination in the corpus callosum. The results of the DTI parameters were strongly correlated with the level of cognitive impairment.

The final way in which DTI has been used to assess AD is WM tractography. A preliminary study in 2006^100^ used DTI imaging to trace WM tracts in patients with AD. This study showed the feasibility of DT-MRI-based tratography in evaluating patients with AD and has been carried out by other labs since then. Now common quantification techniques are applied to DTI in the form of either deterministic and/or probabilistic tractography approaches.^101-106^

DTI has been studied in the setting of differential diagnosis. Zarei et al. found a significant difference in the forceps minor of FA between those with vascular dementia and AD.^101^ A study by Fayed et al. in 2008^102^ found higher values of ADC in Lewy Bodies Disease (LBD) as compared to MCI. A study by Kantarci et al.^84^ noted distinct changes in MD and FA that could be used to differentiate LBD from AD. Chen et al.^91^ compared MD and FA values generated by DTI from those with MCI and various types of dementias. They found that frontotemporal dementia (FTD) subjects showed WM changes in the temporal lobes, anterior subcortical areas, periventricular areas, and in the genu of the corpus callosum. Changes in the corpus callosum genu were seen both in FTD, AD and in subcortical ischemic vascular dementia (SIVD). In that latter changes were also seen in the anterior and posterior periventricular areas and bilateral subcortical areas. This has overlap with what is seen on both subjects with FTD and AD, limiting DTIs ability to differentiate the types of dementias.

**Limitation.** DTI is a promising technique to detect microscopic tissue abnormalities in vivo in early AD. While the details of the different measurements drawn from DTI are under scrutin, It is possible that technique is sensitive to changes such as demyelination, axonal damage, and neuronal loss. The large number of image analysis techniques that can be acquires are one of the confounding factors of DTI interpretation

ROI techniques make a priori judgments on what brain areas to consider based on clinical and pathological knowledge. However, as the underlying pathology of AD is still relatively uncharacterized at the molecular level, using ROI may prove the wrong technique for the elucidation of the first prodromal symptoms of AD. Histogram analysis is an alternative to ROI techniques, which examine a global analysis of brain WM. The major drawback of this technique is limited spatial resolution.^103^ More recently we have seen the emergence of voxel-based analysis, which allows the comparison of DTI parameters to be performed in a normalized space including the whole brain and is independent of a priori assumptions as to the areas that will prove significant. Finally, we have seen the emergence of using DTI tractography to assess WM in AD patients. This technique has the potential to produce information about structural connectivity. Questions still remain as to how tractography can be applied to those with AD.

Using this technique, it is hard to discern MCI from AD. Some researchers have reported differences in WM integrity, but as discussed earlier, there are a lot of potential problems with using measures of integrity to infer structural and functional anatomy. Perhaps in future, by evaluating FA and MD changes in WM, in special in the splenium of corpus callosum, will allow physicians to monitor disease progression with higher efficacy. These findings are, however, tentative because so many conflicting results are present in the literature. Although its correlation between what is observable in the image and the underlying structural and functional histopathology is still under question, DTI tractography has been used with moderate success to differentiate patients with AD from unaffected patients serving as controls. In conclusion, this modality provides much better resolution at a lower level as compared to sMRI. The conclusions derived from these images, however, are still up for debate.

## MAGNETIZATION TRANSFER IMAGING

Microscopic WM changes are also known to be found in AD. Abnormalities in myelin sheaths, axons and oligodendroglia has been reported by Brun et al.^104^ Prevalence estimation of WM hyperintensity (WMH) has ranged from 21-100% in periventricular areas and 32-100% in deep WM regions in patients with AD.^103^ It is controversial the physiopathology role of these WMH in the dementia process and whether they accelerate the cognitive decline in individuals with MCI or not.^106^

Magnetization transfer imaging (MTI) studies in AD have shown significant reduction in magnetization transfer ratio (MTR) in patients as compared with controls in the whole brain,^107,108^ cortical GM, temporal lobes,^81^ and hippocampus.^109,110^ Van der Flier et al.^111^ found decreased peak heights of MTR histograms in MCI and AD as compared to normal controls, reflecting structural brain damage. Mascalchi et al.^110^ reported significantly decreased MTR in the left hippocampus, amygdala, and left posterior medial temporal cortex of patients with AD but no difference was seen between amnestic MCI and controls.

Ridha et al.^107^ found that AD patients had a lower whole brain volume, brain MTR, hippocampal volume, and mean hippocampal MTR compared with the control group. However, of all parameters, only whole brain volume was significantly correlated with cognitive impairment tests. Ropele et al.^112^ examined longitudinal study in 28 patients with mild to moderate AD and 19 controls using MTI at baseline, after 6 and 12 months. At baseline, AD patients showed significant reduction in global MTR when compared with controls. Steady MTR values in AD were seen only in hippocampus but not in striatum and thalamus. However, following up AD patients with MTR might show progressive and constant tissue changes. These global changes were seen already after 6 months paralleled by the regional MTR decreases in structures such as hippocampus, putamen and thalamus. Changes in caudate nucleus were seen only after 12 months. They also found that these MTR changes in hippocampus, putamen, and thalamus were associated with cognitive function that was more pronounced in the left hemisphere.

Ginestroni et al.^113^ has done a volumetric and MTR analysis in 6 carriers of PSEN^1^ mutations compared with 14 healthy subjects. All carriers had a normal daily and working life. One subject in carriers group presented with mild memory deficit, which was confirmed by other family members but did not meet the criteria for probable AD. The investigators reported GM volume and MTR changes in carriers as compared to the control group, more frequent in the temporal lobe. No region showed a correlation between MTR decrease and impairment of cognition, but a slight trend in the temporal lobe. They assumed that non-demented subjects at risk for familial AD may be associated with atrophy and decreased MTR in the temporal cortex.

Studies with quantitative MTI (qMT) have come to in focus of interest. Kiefer et al.^114^ found difference in qMT parameter including T2 of the restricted pool and fractional pool size in the anterior hippocampus could differentiate AD, MCI and health controls. Giulietti et al.^115^ extended the qMT study to the whole brain using a voxel-wise approach. Many brain regions of reduced forward exchange rate were found in AD patients. This study has provided some knowledge to better understand how MT features and forward exchange rate correlates to pathology in AD. Specifically, decrease forward exchange rate might represent a mitochondria functional impairment and that could theoricately enhance the detection of very early signs of neurodegeneration.

Some studies have reported MTR in different types of dementia. Study by Hanyu et al.^109^ found significant lower MTR values in the hippocampus in AD compared to other types of dementia and controls (p<0.001). They reported that MTR analyses were better than visual evaluation of atrophy for differentiating AD from non-AD dementia (an overall discrimination rate of 77% versus 65%). MTR also correlates with MMSE scores and with medial temporal lobe atrophy in AD group but not in patients with non-AD dementia. The same investigating group also found that MTR in the MTL and posterior cingulate WM in both DLB and AD groups were significantly more reduced than those in age-matched controls. MTR in hippocampus of LBD were significantly higher than AD. No difference was found in MTR in the frontal WM among the three groups. For distinguishing DLB from AD, MTR of the hippocampus showed a sensitivity of 76% and specificity of 71%. They suggested that the results may reflect underlying histopathological differences with less severe neuronal degeneration in the hippocampus of DLB.^116^

**Limitation.** MTR is sensitive for microstructural tissue change and able to reveal WM abnormalities that cannot be recognized by conventional MRI. This technique provides high SNR and can be done in a short time period. However, MTR is typically measured in a region of interest and thus only part of the brain is analyzed. The results depend on how and where the regions of interest are drawn. Histograms may be used as an alternative for analysis of entire MTR data set. This method may introduce a bias because the MT data of both GM and WM are analyzed together. A change in GM-to-WM ratio leads to a change in the MTR histogram that is the unrelated to a true change in the MTR. The use of MTR in AD patients is still limited. The finding of decreased MTR referring to demyelination and axonal loss is not exclusively related to AD. This finding is also found in other pathological process such as inflammation. Several factors have been shown to affect MTR value, including the repetition time, echo time, the hardware and many other technical causes. For now, MTR has not yet appeared to be able to contribute to AD management nor will it likely gain a role in clinical practice.
